# Optimal presentation of decision explanations: investigating the effects of explainability and reliability on medical diagnostic performance with conversational AI

**DOI:** 10.3389/fpsyg.2026.1816652

**Published:** 2026-09-24

**Authors:** Xin Chu, Fangyuan Chen, Mu Tong, Yun Lu

**Affiliations:** 1 Department of Thoracic Surgery, Zhongda Hospital, Southeast University, Nanjing, China; 2 Department of Cardiology, The Second Affiliated Hospital of Xuzhou Medical University, Xuzhou, China; 3 School of Design, South China University of Technology, Guangzhou, China; 4 Department of Cardiac Surgery, XuZhou Central Hospital, Southeast University, Nanjing, China

**Keywords:** design for AI, ergonomics, explainable artificial intelligence, human-computer interaction (HCI), information visualization

## Abstract

**Introduction:**

The effective and efficient presentation of decision-making rationales within artificial intelligence black boxes remains a critical factor limiting the application of conversational AI in medical diagnosis. This study investigated the effects of decision explanation formats and system reliability on physicians’ diagnostic behavior.

**Methods:**

A simulated diagnostic experiment involving 22 clinicians identifying complex pulmonary conditions was conducted. Four explanation formats and two levels of system reliability were manipulated. Diagnostic accuracy, task completion time, subjective cognitive load, trust ratings, and user preferences were measured.

**Results:**

Explanation format did not significantly affect diagnostic accuracy but significantly affected task completion time, subjective cognitive load, and trust ratings. A significant interaction between explanation format and system reliability was observed for task completion time. Decision tree explanations yielded the lowest cognitive load, whereas flowcharts resulted in the longest task completion times across reliability conditions. Highlighted text received the highest trust ratings and subjective preference scores. System reliability and explanation format played complementary roles in human–AI collaborative diagnosis, with the efficiency of different explanation formats varying across reliability conditions.

**Discussion:**

These findings highlight the joint roles of decision explanation format and system reliability in conversational AI and provide empirical evidence for the design and optimization of medical AI interfaces.

## 1 Introduction

Conversational artificial intelligence (AI), as a form of AI capable of simulating natural human-language interaction, has been increasingly applied across a wide range of domains with the rapid development of Large Language Models (LLMs), including medical consultation, educational tutoring, industrial operations, and even military decision-making (Tu et al., 2024; Milne-Ives et al., 2020; Kiangala and Wang, 2025; Páleník et al., 2025; Khosravi et al., 2022; Ji et al., 2023). Owing to its interaction style that closely resembles human communication and its relatively low learning cost, existing research generally suggested that conversational AI is likely to become one of the dominant deployment paradigms of AI systems in the future (Gottlieb et al., 2023; Kulkarni et al., 2019).

In the medical domain, conversational AI has gradually been introduced into multiple stages of clinical practice. These applications included assisting primary care physicians in basic disease assessment (Cho et al., 2025), supporting specialist clinicians in complex diagnostic decision-making (Tu et al., 2025) and exploring its potential use in preoperative planning (Dwyer et al., 2023). In addition, conversational AI has been applied in medical education and training, as well as in long term health management contexts (Mayta-Tovalino et al., 2024; Mahajan and Powell, 2025). Prior studies had demonstrated that conversational AI may play a significant role in alleviating physicians’ workload, compensating for limited clinical experience, and enhancing diagnostic decision-making performance, particularly for clinicians with less experience and for diagnostic scenarios associated with a high risk of misdiagnosis (Gandhi et al., 2023; Jacobs et al., 2025; Lin et al., 2025).

However, in diagnostic contexts involving complex diseases, the practical deployment of conversational AI still faced substantial challenges, among which limited understanding of and trust in AI-generated diagnostic conclusions remained one of the most prominent barriers (Ennab and Mcheick, 2024). In clinical practice, even when AI systems were capable of providing professionally sound and logically coherent diagnostic recommendations, physicians may choose to disregard or cautiously adopt these outputs if the underlying decision rationale cannot be adequately understood. This issue largely stemmed from the insufficient transparency of the decision-making processes of existing AI systems, which made their reasoning logic difficult for human users to comprehend and verify within limited time constraints. Previous studies have referred to this property as AI explainability, which was generally defined as the ability of an AI system to present its decision rationale and reasoning process in a form that can be understood by human users (Kovari, 2025; Jentzsch et al., 2019).

Recently, research on AI explainability has increased steadily. In the medical domain in particular, explainability was widely regarded as a key factor influencing physicians’ trust in, and willingness to adopt AI systems (Frasca et al., 2024). Compared with AI systems that only provide predictive outputs, systems that accompany diagnostic results with explanatory information are more likely to gain physicians’ trust (Rezaeian et al., 2026). Previous studies have proposed a variety of explanation approaches to address the black-box nature of AI systems. These approaches were commonly categorized into local explanation methods, such as Local Interpretable Model-agnostic Explanations (LIME), and global explanation methods, such as SHapley Additive exPlanations (SHAP) (Band et al., 2023; Zacharias et al., 2022; Vimbi et al., 2024). For example, when AI systems provide diagnostic recommendations together with visual explanations based on feature importance, physicians tend to report higher levels of trust and satisfaction (Vimbi et al., 2024). In addition, when AI systems adopt example-based explanations by presenting historical cases similar to the current case, physicians show significant improvements in both diagnostic accuracy and acceptance of AI systems (Ennab and Mcheick, 2022; Kamath and Liu, 2021).

Although previous studies have demonstrated the positive role of explanation mechanisms in improving physicians’ diagnostic performance and understanding of decision-making, some research has largely focused on the presentation of local information or isolated cues. As a result, their applicability has mainly been demonstrated in task scenarios that are relatively simple in structure or involve well defined decision pathways. In the diagnosis of complex and challenging diseases, however, physicians often need to perform integrative reasoning over multidimensional information provided by AI systems. This process went beyond assessing the importance of individual features and requires understanding and integrating relationships across different levels of information. More importantly, prior research indicated that the effectiveness of AI explainability depends to a large extent on its interaction design at the human machine interface level (Liao et al., 2020; Abdul et al., 2018), which places higher demands on the visual organization of explanatory information.

In response to this issue, existing studies have explored different forms of explanatory information, including tables, causal diagrams, rich text, and keyword highlighting, and have identified the respective advantages and applicability boundaries of these visualization approaches across different contexts (Reyes et al., 2020; Ashktorab et al., 2019; Muralidhar et al., 2025; Ahn et al., 2024; Schoenherr et al., 2023). However, some researchers pointed out that different usage scenarios require different types and depths of explanations (Liao et al., 2020; Razzaki et al., 2018). In particular, when AI systems were used to support complex medical diagnostic tasks, especially those involving multiple potential etiologies and highly similar imaging features, there was still a lack of systematic empirical evidence on how explanatory information should be organized and visualized in order to effectively support physicians’ understanding of the overall diagnostic logic of AI systems. This gap constituted the central focus of the present study.

Beyond explainability, the reliability of AI systems was also widely regarded as another critical factor influencing physicians’ trust and usage behavior (Razzaki et al., 2018; Asan et al., 2020). Reliability typically referred to the accuracy and consistency of an AI system’s output across different situations (Ryan, 2020). Existing research has shown that users’ trust in automated systems was highly dependent on system reliability; once errors occur, trust levels tend to decline rapidly, and even when system performance subsequently stabilizes, trust was often difficult to fully restore (Centeio Jorge et al., 2023; Madhavan et al., 2006). In the medical domain, this phenomenon was particularly pronounced. Related studies have also found that physicians and patients generally exhibit lower tolerance for errors made by AI systems than for errors made by human physicians, a phenomenon commonly referred to as algorithm aversion (Jones-Jang and Park, 2022; Burton et al., 2020). Consequently, the reliability of AI systems in medical diagnosis largely determined whether they can be used on a sustained basis. However, the relationship between reliability and explainability was not a simple linear one. On the one hand, AI systems with higher reliability were more likely to establish physicians’ initial trust. On the other hand, well-designed explainability mechanisms may partially compensate for trust loss resulting from insufficient system reliability (Ryan, 2020; Hu et al., 2019; Zerilli et al., 2022). Accordingly, it remains insufficiently understood whether, and how the effectiveness of different explanation presentation formats changes as AI reliability varies. This constitutes the second research gap addressed in the present study.

Based on the above research background, the present study designed a simulated experiment using a typical differential diagnosis task of pulmonary lesions as the diagnostic scenario, and examined how different AI explanation formats, under varying levels of system reliability, influence physicians’ task performance and subjective attitudes. The aim was to characterize the features of explanation formats in the context of complex diagnostic tasks, and to elucidate the mechanisms through which reliability and explainability jointly affect human to AI collaborative performance, thereby providing empirical evidence for the design and application of conversational AI systems in clinical diagnostic support. Based on these objectives, the following hypotheses were proposed.

H1: AI explanation formats significantly affect diagnostic performance; decision trees will yield the highest accuracy and shortest diagnostic time.

H2: Changes in explanation formats will significantly influence users’ subjective cognitive load. Tabular explanations will be associated with the lowest cognitive load, whereas flowchart explanations will result in the highest cognitive load.

H3: Regarding result credibility, decision tree explanations will receive the highest scores, while highlighted text will receive the lowest.

H4: There is an interaction between AI reliability and explanation format; specific formats can improve collaborative diagnostic performance under low-reliability conditions.

H5: Regarding user preference, decision trees will be more favored due to their efficient presentation of logical reasoning.

Because some hypotheses contained multiple predefined predictions, each hypothesis was evaluated as a whole. A hypothesis was considered supported only when all predictions explicitly specified within that hypothesis were satisfied; otherwise, it was considered not supported.

## 2 Methods

### 2.1 Experimental design

The study employed an 4 × 2 within-subjects design. The independent variable A was the decision explanation format, including four conditions: decision tree, flowchart, table, and highlighted text, as shown in Figure 1. These four formats were selected because they represent distinct strategies for organizing explanatory information that are relevant to complex diagnostic reasoning. Decision trees emphasize hierarchical and branching relationships, flowcharts emphasize procedural and causal relationships, tables facilitate categorical comparison, and highlighted text preserves contextual information while emphasizing key evidence. These formats therefore provided contrasting representations of hierarchical, causal, categorical, and contextual information (Liu and Salvendy, 2007; Khan and Khan, 2011; Friendly, 2001; Strobelt et al., 2016). The independent variable B was the reliability level of the conversational AI, categorized into high and low reliability, which was manipulated by setting different frequencies of incorrect diagnostic suggestions during the experiment.

**Figure 1:**
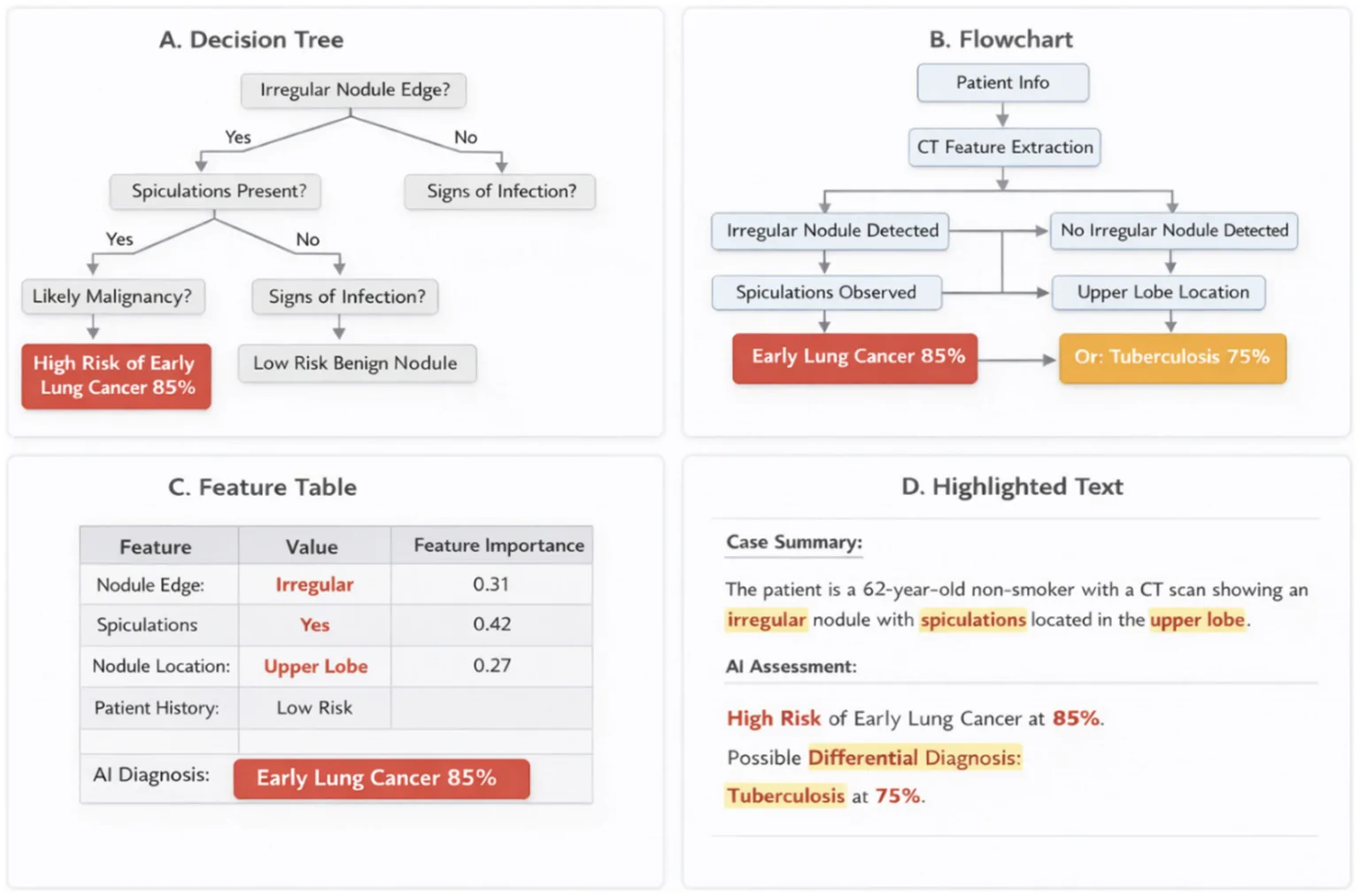
Visualization formats of decision explanations. **(A)** Decision tree; **(B)** Flowchart; **(C)** Table; **(D)** Highlighted text.

The complex pulmonary disease diagnosis tasks were selected as the experimental scenario, specifically simulating the diagnostic process in clinical practice that requires reasoning and judgment based on the synthesis of multi-source information. Specifically, the experimental task revolved around the comprehensive differential diagnosis of pulmonary lesions, requiring doctors to judge potential disease types based on patient history, symptoms, laboratory test results, and imaging features. Such diagnostic tasks typically involved distinguishing between neoplastic and infectious lesions, such as early-stage lung cancer, tuberculosis, and inflammatory lesions, which have significant overlap in clinical manifestations and imaging features, making the diagnostic process markedly uncertain and complex in reasoning (Mazzone and Lam, 2022; Ost and Gould, 2012). Furthermore, diagnosing such diseases usually required doctors to integrate multi-source patient information and perform an exclusionary process to form a final diagnostic opinion through comprehensive judgment. This involved complex multi-path identification and decision-making processes (Parker et al., 2018) which were challenging for inexperienced doctors and represent a suitable application scenario for AI intervention in clinical settings. In the experiment, participants were required to make diagnostic decisions after reading case information, combined with the diagnostic suggestions provided by the conversational AI.

To comprehensively assess participants’ behavioral performance and subjective perceptions, the study collected both diagnostic performance metrics and subjective evaluation measures. Diagnostic performance included the task completion time and diagnostic accuracy. For subjective evaluation, cognitive load under different explanation visualization conditions was assessed using the NASA-TLX method (Hart and Staveland, 1988), while participants’ trust in the AI system was measured using the TrAAIT model based on a 5-point Likert scale (Stevens and Stetson, 2023; Bauer and Michalowski, 2025). All questionnaires were created and administered via the Questionnaire Star platform. Finally, participants’ subjective preferences for different explanation visualization formats were collected through direct questioning at the end of the subjective evaluation session (Muralidhar et al., 2025). These dependent measures were specified *a priori* to capture distinct and complementary aspects of human to AI collaboration, including diagnostic accuracy, collaborative efficiency, cognitive demand, and subjective trust, rather than as interchangeable measures of a single outcome.

### 2.2 Prototype design

An experimental prototype interface was constructed based on the Docus.ai system, as shown in Figure 2. This system is utilized in commercial settings for computer-aided medical diagnosis, allowing physicians to select specific patients and input corresponding symptom information to generate matched diagnostic outcomes and recommended actions (Muralidhar et al., 2025; Shin, 2021). By embedding four decision explanation schemes into the prototype interface, a visual representation of the AI’s decision-making process could be presented to the participants. Specifically, the prototype interface was divided into several information zones, including a case information area, a dialogue display area, and an information input area. Once a user clicked the blue button at the top right of the AI decision, the interface transitioned to the decision explanation page, as shown in Figure 3. On this page, consistent with the experimental design, the decision explanation formats were presented as tabular explanations, decision tree explanations, flowchart explanations, and highlighted text explanations.

**Figure 2:**
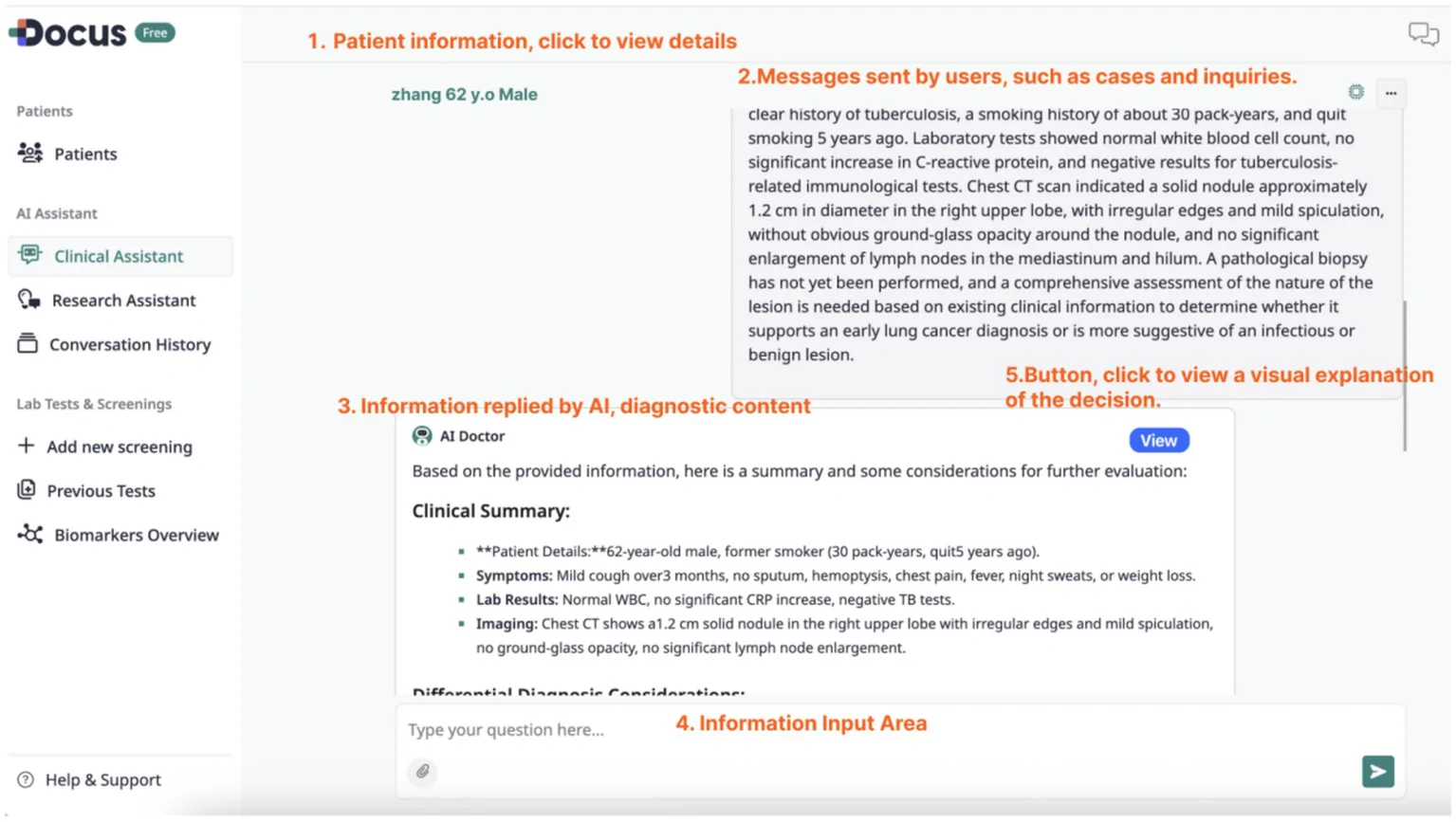
User interface prototype used in the experiment.

**Figure 3:**
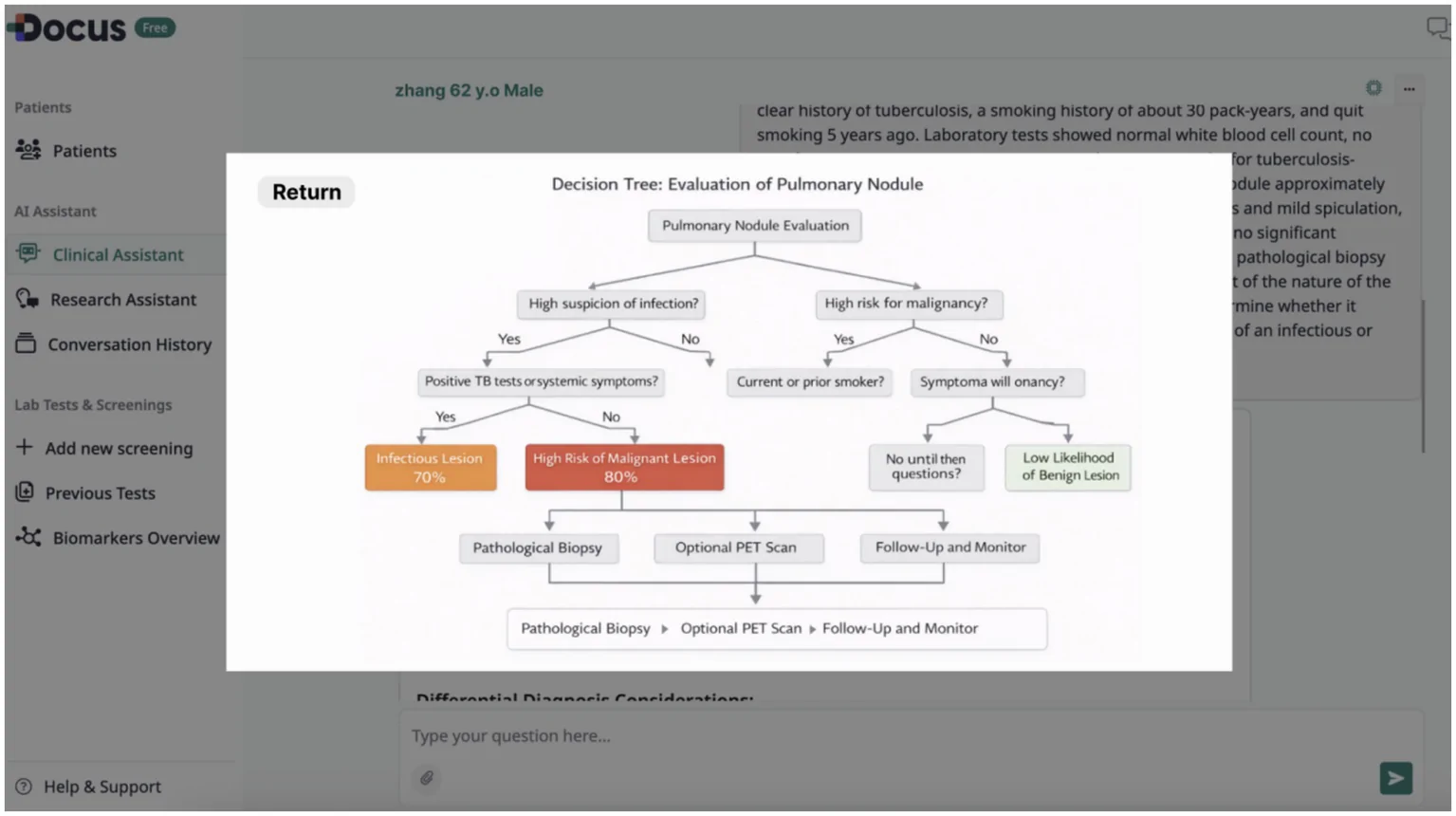
Pop-up window for displaying explainability information.

### 2.3 Participants

A total of 22 general practitioners were recruited through online channels to participate in the experiment. An *a priori* power analysis was conducted using G*Power 3.1 for a repeated-measures ANOVA. Assuming a medium effect size (*f* = 0.25), α = 0.05, and power = 0.80, the minimum required sample size was 16 participants. Most participants were from community hospitals and large general hospitals. Participants ranged in age from 27 to 39 years (M = 32.6, SD = 4.43), and all had more than 2 years of clinical experience. All participants reported no history of psychiatric disorders and had normal or corrected-to-normal vision. All participants provided informed consent and were informed that the purpose of the study was to evaluate a prototype interface of an AI-based medical assistant.

### 2.4 Experimental procedure

A practice session was conducted prior to the formal experiment, during which the researchers explained the precautions and basic operations to the participants. Subsequently, participants were required to use Docus.ai to enhance their contextual understanding of collaborative diagnosis, as shown in Figure 4. The software was loaded with data from 42 clinical cases featuring similar chest lesion characteristics; participants were required to perform diagnoses for two designated cases.

**Figure 4:**
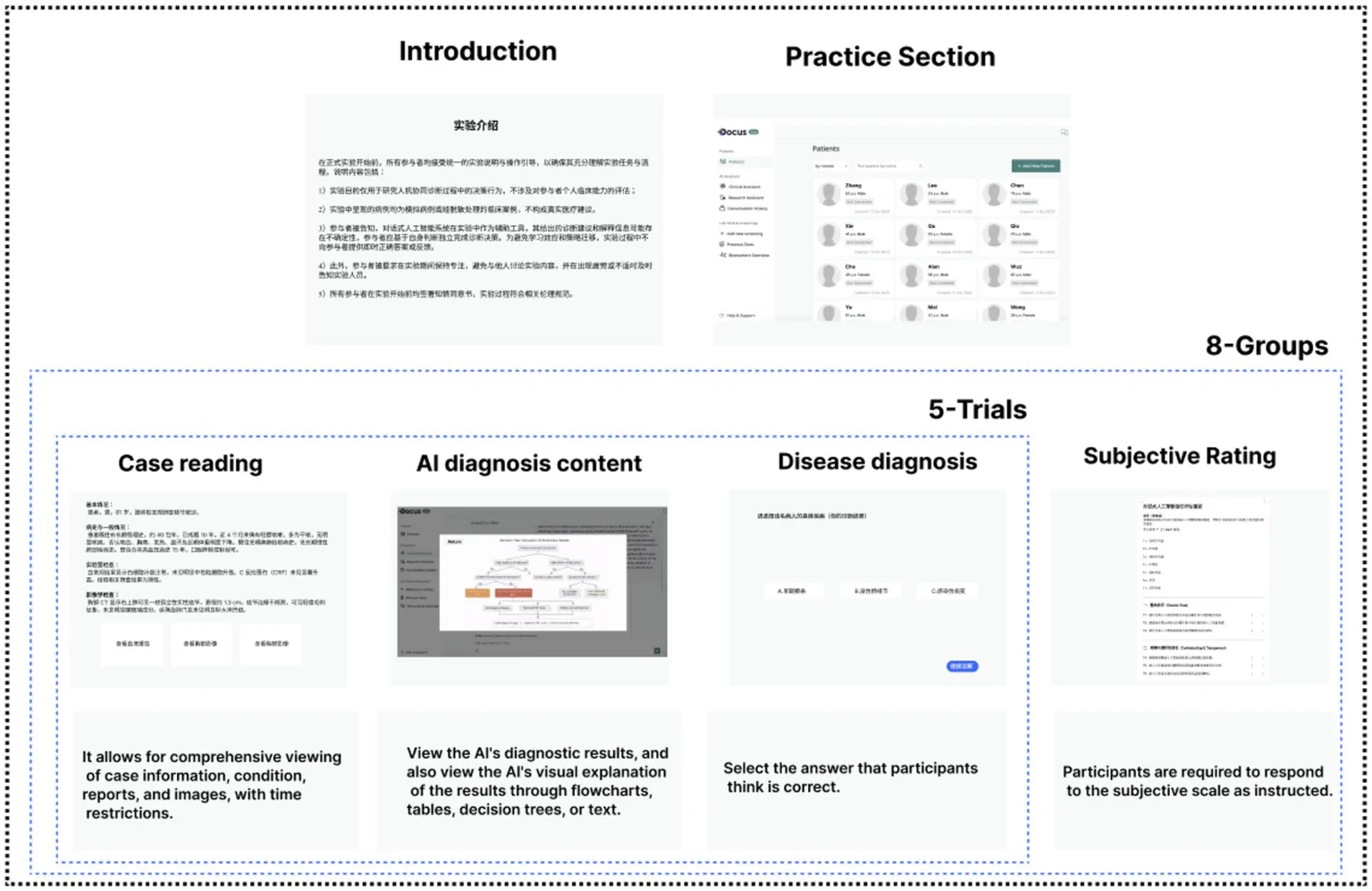
Overview of the experimental procedure.

The formal experiment followed the practice session and consisted of 8 experimental blocks based on the predefined conditions. The presentation order of the eight experimental blocks was counterbalanced across participants to minimize potential practice and order effects. Within each block, the five diagnostic trials were presented in a randomized order. Participants completed diagnoses for 5 patients, resulting in a total of 40 trials per participant. In a single trial, participants first reviewed the patient’s clinical materials within a 90-s time limit, after which the system transitioned to the AI-assisted diagnostic interface. This interface presented the AI’s diagnostic results and recommended actions. Participants were instructed to review these contents and examine the explanations of the diagnostic process to confirm the medical condition. Upon making a diagnostic judgment, participants clicked a button to proceed to the response interface. There, they selected the correct answer from three options and clicked a button to continue the next case. After completing each experimental block, participants filled out subjective scales and were allowed a break for 3 min. Cash compensation was provided to all participants upon completion of the entire experiment.

### 2.5 Data analysis

A total of 22 datasets were collected, comprising both behavioral and subjective data. The collected data and their processing procedures are described as follows:

Diagnostic Accuracy (DA), for each diagnostic trial, participants selected one diagnosis from three alternatives. Each response was coded as correct (1) or incorrect (0), and diagnostic accuracy was calculated as the proportion of correct responses under each experimental condition.
Task Completion Time (TCT), Task completion time was automatically recorded by the experimental system from the beginning of the AI-assisted diagnostic task to the submission of the participant’s final diagnostic response. The raw timing data were recorded in milliseconds and converted to seconds for subsequent analysis.
Subjective Cognitive Load (SCL): Subjective cognitive load was assessed using the NASA-TLX after each experimental condition, and the resulting score was used to represent participants’ perceived cognitive load during the diagnostic task.
AI Trust Rating (ATR): Participants’ trust in the conversational AI system was assessed using the TrAAIT scale based on a 5-point Likert response format, with the resulting score representing the participant’s perceived trust in the AI system.
Subjective Preference: After completing all experimental conditions, participants were asked to select their preferred decision explanation format and briefly report the reasons for their choice.

Given the within-subjects design of the present study, all participants completed the diagnostic tasks under different decision explanation formats and AI reliability conditions. Therefore, repeated-measures analyses of variance were conducted separately for the behavioral and subjective measures. Decision explanation format and AI reliability were treated as within-subject factors. Task completion time and diagnostic accuracy during the diagnostic decision stage were analyzed separately to evaluate the effects of the experimental conditions on human-AI collaborative diagnostic performance. Similarly, subjective cognitive load and trust ratings toward the conversational AI system were analyzed separately to examine the effects of the experimental conditions on participants’ subjective experience.

Prior to the ANOVAs, the distributions of the dependent variables were examined using Shapiro–Wilk tests in conjunction with Q-Q plots, and extreme values were screened and, where necessary, appropriately handled. For within-subject effects involving more than two levels, the sphericity assumption was assessed using Mauchly’s test. The significance level was set at 0.05. When a significant main effect involving more than two levels was identified, pairwise comparisons among the relevant experimental conditions were conducted using Bonferroni adjustment for multiple comparisons. Significant interaction effects were further examined using simple-effects analyses, with Bonferroni follow-up comparisons where appropriate.

## 3 Results

The normality assessment revealed no substantial deviations from normality for the dependent measures used in the subsequent analyses (Shapiro–Wilk tests, all *p* > 0.05), which was also supported by inspection of the Q-Q plots. During data screening, three exceptionally long TCT observations (>300 s) were identified. These observations were considered to reflect temporary task disengagement and were replaced with the mean TCT of the remaining valid trials from the same participant under the same experimental condition. No participants were excluded, and no notable outliers requiring treatment were identified in the subjective measures. Mauchly’s tests did not indicate violations of the sphericity assumption for the relevant within-subject effects (all *p* values > 0.05); therefore, no Greenhouse–Geisser correction was required.

### 3.1 Task accuracy

The descriptive statistics of diagnostic accuracy were shown in Table 1. The results indicated that the explanation formats had no significant effect on participants’ diagnostic accuracy, *F* (3, 63) = 1.026, *p* > 0.05. In contrast, AI reliability had a significant main effect on diagnostic accuracy, *F* (1, 21) = 12.571, *p* < 0.05. The interaction effect between explanation formats and reliability was not significant, *F* (3, 63) = 0.957, *p* > 0.05. Further analyses indicated that diagnostic accuracy under the high reliability condition (M = 0.975, SD = 0.042) was significantly higher than that under the low reliability condition (M = 0.955, SD = 0.068).

**Table 1:** Descriptive statistics of task performance data.

Factor	Level	Accuracy		Task completion time		Subjective cognitive load		Trust rating	
		Mean	SD	Mean	SD	Mean	SD	Mean	SD
Explanation format	Decision tree	0.973	0.046	93.02	18.65	6.87	1.03	4.05	0.78
	Flowchart	0.955	0.051	105.67	25.01	8.35	1.49	3.53	0.69
	Table	0.968	0.048	88.17	16.99	7.34	1.32	3.02	0.82
	Highlighted text	0.964	0.049	91.71	17.26	7.92	1.58	4.17	1.03
AI reliability	High	0.975	0.042	87.59	19.60	7.95	2.96	4.66	1.10
	Low	0.955	0.068	101.70	21.39	7.28	2.62	2.72	1.72

### 3.2 Task completion time

The ANOVA results revealed a significant main effect of explanation formats on task completion time, *F* (3, 63) = 9.879, *p* < 0.001. A significant main effect of AI reliability was also observed, *F* (1, 21) = 5.653, *p* < 0.05. In addition, the interaction effect between explanation formats and reliability on task completion time was significant, *F* (3, 63) = 5.054, *p* < 0.05.

To further examine this interaction, simple-effects analyses were conducted in conjunction with the interaction plot shown in Figure 5. Bonferroni-adjusted pairwise comparisons showed that, under the low-reliability condition, task completion time in the flowchart condition (M = 112.56, SD = 26.32) was significantly longer than that in the decision-tree (M = 107.03, SD = 24.08; MD = 5.53 s, *p* = 0.034), tabular (M = 91.14, SD = 15.63; MD = 21.42 s, *p* < 0.001), and highlighted-text conditions (M = 96.08, SD = 15.40; MD = 16.48 s, *p* < 0.001). Under the high-reliability condition, task completion time in the flowchart condition (M = 98.78, SD = 18.59) was also significantly longer than that in the highlighted-text (M = 87.34, SD = 14.29; MD = 11.44 s, *p* < 0.001), decision-tree (M = 79.01, SD = 16.36; MD = 19.77 s, *p* < 0.001), and tabular conditions (M = 85.21, SD = 17.43; MD = 13.57 s, *p* < 0.001). Among the four formats, the decision tree condition yielded the shortest task completion time under high reliability.

**Figure 5:**
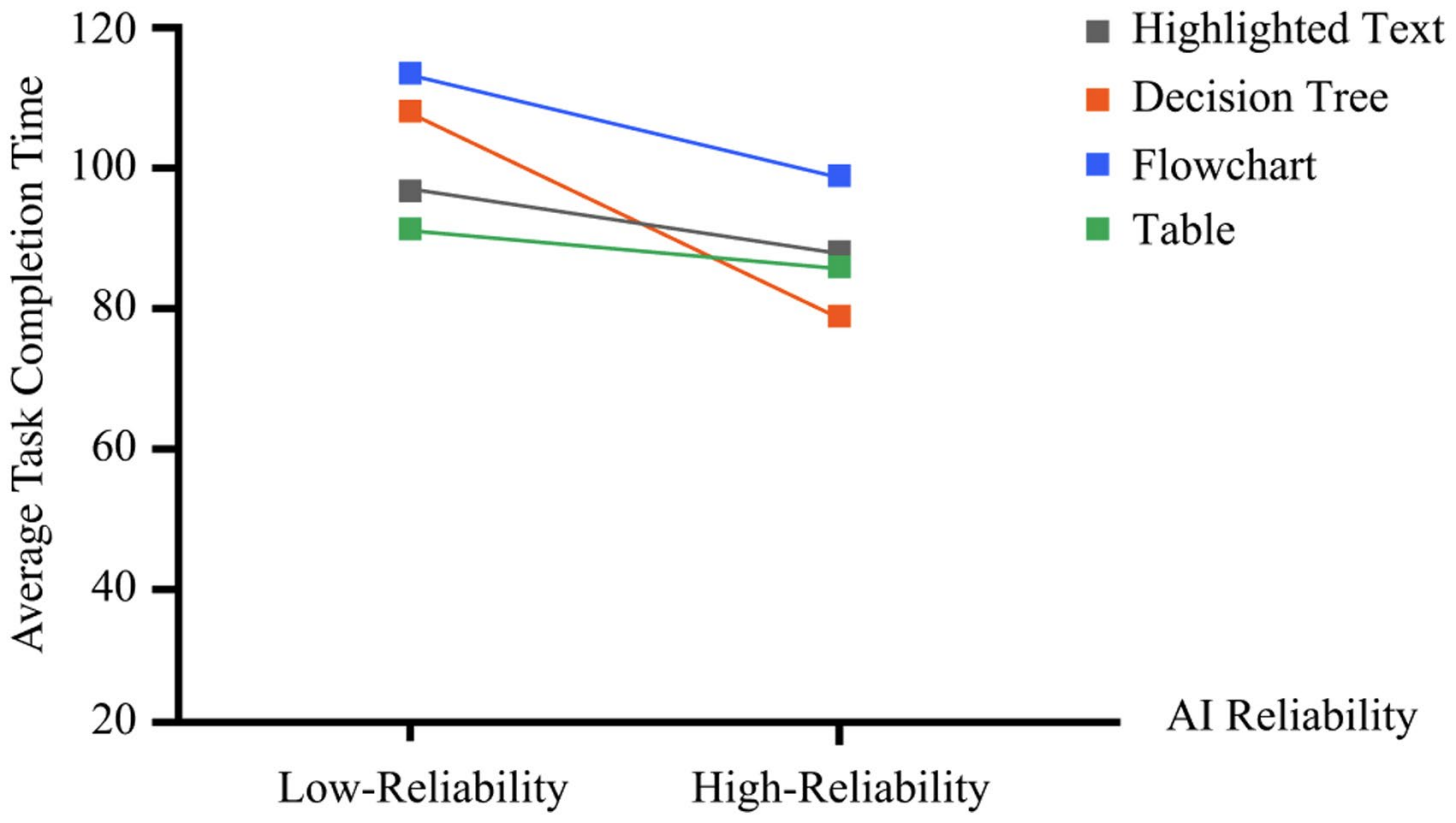
Interaction plot of task completion time.

Overall, the results indicated that, regardless of reliability level, decision explanations presented in a flowchart format consistently resulted in the longest task completion times.

### 3.3 Subjective cognitive load

The results indicated a significant main effect of explanation formats on participants’ subjective cognitive load, *F* (3, 63) = 17.011, *p* < 0.001. In contrast, the main effect of AI reliability on subjective cognitive load was not significant, *F* (1, 21) = 0.774, *p* > 0.05. No significant interaction effect between explanation formats and reliability was observed for subjective cognitive load, *F*(3, 63) = 0.906, *p* > 0.05.

*Post hoc* comparisons revealed that subjective cognitive load under the decision tree condition (M = 6.87, SD = 1.03) was significantly lower than that under the highlighted text (M = 7.92, SD = 1.58, *p* = 0.003), tabular (M = 7.34, SD = 1.32, *p* = 0.018), and flowchart conditions (M = 8.35, SD = 1.49,*p* < 0.001).

### 3.4 Trust rating

A 4 × 2 ANOVA was conducted on trust ratings, and the results were summarized in Table 2. The analysis showed a significant main effect of explanation formats on trust, *F* (3, 63) = 4.636, *p* < 0.05. A significant main effect of AI reliability on trust was also observed, *F* (1, 21) = 19.862, *p* < 0.001. No significant interaction effect between explanation formats and reliability was found for trust rating, *F* (3, 63) = 0.534, *p* > 0.05.

**Table 2:** Summary of ANOVA results for behavioral and subjective metrics.

Effect	Diagnostic accuracy			Task completion time			Subjective cognitive load			Trust rating		
	F	*p*	η_p_^2^	F	*p*	η_p_^2^	F	*p*	η_p_^2^	F	*p*	η_p_^2^
XAI	1.026	>0.05	0.047	9.879	<0.001	0.320	17.011	<0.001	0.447	4.636	<0.05	0.181
RL	12.571	<0.05	0.374	5.653	<0.05	0.212	0.774	>0.05	0.036	19.862	<0.001	0.486
Int	0.957	>0.05	0.044	5.054	<0.05	0.194	0.906	>0.05	0.041	0.534	>0.05	0.025

XAI, explanation format; RL, AI reliability; Int, interaction between explanation format and AI reliability.

*Post hoc* comparisons indicated that the highlighted text condition (M = 4.17, SD = 1.03) yielded significantly higher trust ratings than the table (M = 3.02, SD = 0.82, *p* < 0.001), flowchart (M = 3.53, SD = 0.69, *p* < 0.001), and decision tree (M = 4.05, SD = 0.78, *p* = 0.023) conditions. In addition, trust ratings under the high reliability condition (M = 4.66, SD = 1.10) were significantly higher than those under the low reliability condition (M = 2.72, SD = 1.72).

### 3.5 Subjective preference

In addition to behavioral and subjective rating measures, participants’ subjective preferences for various decision explanation formats were recorded. Among the 22 participants, highlighted text emerged as the most favored format, chosen by 8 individuals. This was followed by decision trees, which were selected by 5 participants. Tables and flowcharts were preferred by 3 and 2 participants, respectively. The remaining 4 individuals reported no distinct preference, stating they were unable to select a single optimal format.

To further understand these choices, participants’ qualitative feedback was aggregated, with the primary rationales for each selection summarized in Table 3.

**Table 3:** Participants’ subjective preferences for explanation formats.

Explanation format	Number of participants	Reasons
Flowchart	2	1) Clearly displayed the overall diagnostic decision-making flow, aiding in understanding the sequence of steps. 2) Aligned with the step-by-step screening mindset used in clinical diagnosis, making it easy to follow the reasoning process.
Table	3	1) Provided an intuitive comparison of different diagnostic features and corresponding information, allowing for rapid review of key indicators. 2) Information presentation was concise and clear, facilitating quick access to core factors underlying the conclusion.
Decision tree	5	1) Clearly illustrated the hierarchical relationships between different judgment criteria during the diagnostic process. 2) The conditional branching format can visualize diagnostic logic, helping to form a clearer basis for decision-making. 3) Helped in understanding how the AI narrows down the diagnostic scope to reach a conclusion. 4) Presented information modules coherently, reducing the cognitive load caused by switching between different information blocks.
Highlighted text	8	1) Most familiar format as it was the most common in current conversational AI, leading to lower understanding and learning burdens. 2) Highlighting key information helped in quickly grasping critical diagnostic points. 3) Preserved the complete diagnostic context and materials, which better aligns with actual clinical reading habits.
Unable to choose	4	1) Different formats have distinct advantages in various aspects, making it difficult to differentiate their pros and cons. 2) Subjective preference was heavily influenced by personal reading habits, making it hard to choose a single option.

## 4 Discussion

Using pulmonary disease diagnosis as the task domain, this study conducted a simulated cognitive experiment involving 22 clinicians using a prototype conversational AI system. We systematically investigated the effects of decision explanation format and system reliability on physicians’ diagnostic performance and subjective evaluations. The findings showed that decision explanation format was significantly associated with task completion time, subjective cognitive load, and trust, whereas no statistically significant effect was detected for diagnostic accuracy. A significant interaction between Explanation Format and AI Reliability was observed specifically for task completion time. Among the different explanation formats, decision trees yielded the lowest cognitive load, whereas flowcharts resulted in the highest. Highlighted text elicited the highest subjective trust ratings and was also the most frequently preferred explanation format, selected by 36% of participants. According to the predefined criterion that all constituent predictions within a hypothesis had to be satisfied, H1-H5 were not supported as originally formulated, although several of the specific effects anticipated by these hypotheses were observed.

Taken together, these findings suggest that the effectiveness of decision explanation formats in conversational AI is related to the organizational structure of the presented information, users’ allocation of cognitive resources, and the reliability level of the AI system. When designing decision explanation formats for user interfaces, designers should note that providing more decision-related information is not necessarily beneficial, nor is a more strongly interconnected information structure necessarily preferable. Instead, the organization and presentation of explanatory information should be matched to the characteristics of the diagnostic task and the broader interaction context. A detailed discussion of these findings follows.

### 4.1 Differential performance of decision explanation formats

First, in complex medical diagnostic scenarios, different decision explanation formats were associated with distinct patterns of behavioral and subjective responses, which is broadly consistent with previous findings in industrial, autonomous driving, and general medical diagnostic contexts (Ennab and Mcheick, 2024; Frasca et al., 2024; Hudon et al., 2021). In the present study, the decision tree condition was associated with relatively lower subjective cognitive load and, under the high-reliability condition, the shortest task completion time. Its clear hierarchical branching structure may have facilitated the identification of diagnostic pathways and reduced the need to switch repeatedly between different information elements. This interpretation is also consistent with previous research showing that hierarchical visualization can facilitate information processing and reduce cognitive demand in complex tasks (Bogdanov et al., 2025; Padilla et al., 2018).

In contrast, the flowchart condition was associated with higher subjective cognitive load and longer task completion time. One possible explanation is that its larger number of information nodes and paths increased the amount of information that participants needed to process. The tabular format showed relatively favorable performance in task completion time and cognitive load, possibly because its parallel presentation structure facilitated rapid information comparison, as also suggested in previous studies (Huang et al., 2009; Johnson, 2020). However, tabular explanations lacked a coherent decision flow, which may require additional integration during logical reasoning. Highlighted text, meanwhile, received relatively high trust ratings and subjective preference, potentially reflecting participants’ familiarity with continuous clinical text and keyword highlighting. However, the presentation of more complete contextual information may also increase reading and information-processing demands, which may be reflected in the relatively higher cognitive load observed in this condition (DeStefano and LeFevre, 2007; Eppler and Mengis, 2004).

No statistically significant effect of decision explanation format on diagnostic accuracy was detected under the present experimental conditions. This non-significant finding should be interpreted cautiously, particularly given the relatively high overall diagnostic accuracy and the constrained three alternative response format used in this study. Previous studies have reported varying effects of explainability on diagnostic decision-making (Erasmus et al., 2021; Xu et al., 2023). Rather than indicating a direct inconsistency between the present findings and previous studies, such variations may reflect differences in task difficulty, participant expertise, response format, and experimental context. In the present study, all participants had clinical experience, while the explanation formats altered the organization and presentation of information without introducing additional diagnostic evidence. These characteristics may have contributed to the limited variation observed in diagnostic accuracy.

### 4.2 The role of system reliability and its interaction

System reliability showed significant effects on diagnostic accuracy, task completion time, and trust ratings. Diagnostic accuracy and trust ratings were significantly higher under the high-reliability condition than under the low-reliability condition, which is consistent with previous findings in the field of human-AI trust suggesting that system reliability is an important factor influencing user performance and subjective trust (Wright et al., 2020; Gebru et al., 2022).

More importantly, a significant interaction between AI reliability and explanation format was observed specifically for task completion time. This finding indicates that the efficiency associated with different explanation formats varied depending on the reliability level of the AI system. Under low-reliability conditions, the longer task completion times observed for some explanation formats may reflect additional verification and reflective processing when physicians evaluated AI recommendations (Dietvorst et al., 2015; Park, 2014). Such additional processing may partly explain why the efficiency associated with different explanation formats varied under changes in system reliability. Conversely, under high-reliability conditions, users may have required less verification of AI recommendations, allowing the differences among explanation formats to be reflected more directly in task completion efficiency (Hoff and Bashir, 2015).

Taken together, these findings suggest that reliability and explanation format may play complementary roles in human-AI collaborative diagnosis. Reliability was associated with differences in diagnostic performance and trust, whereas the significant interaction observed for task completion time indicates that the efficiency of different explanation formats may also depend on the reliability of the AI system. Thus, the effectiveness of explanation design should be considered in conjunction with system reliability rather than as an independent property of the interface.

### 4.3 Implications for interface design

The findings of this study provided direct implications for the design of decision explanation interfaces in medical AI systems. First, in diagnostic tasks requiring complex logic and multi-step screening, the decision tree format, with its clear hierarchical structure and progressive logical paths, effectively reduced cognitive load and enhanced human-AI collaborative efficiency. Consequently, it was highly suitable as the core view for decision explanations. Second, the highlighted text format performed best in terms of subjective trust and user preference. This indicated that physicians are more receptive to explanatory methods that align with their established clinical reading and reasoning habits, offering contextual continuity and complete information. Based on this, designers should consider integrating structured decision tree explanations with text highlighting. For example, textual descriptions of key evidence could be presented alongside the tree based logical structure, or the interface could allow users to toggle flexibly between structured and textual views to achieve a balance between decision efficiency and subjective trust. In contrast, table formats were better suited as supplementary rapid comparison tools for presenting feature weights or key information summaries, rather than serving as the primary explanatory component. Flowcharts should be used with caution in interface design, as they introduced significant cognitive load and time costs in this study. In scenarios where process-oriented expression was unavoidable, complexity should be controlled by reducing the number of branching paths and compressing hierarchical depth.

Overall, the design of decision explanation interfaces in medical AI systems should not seek a single optimal presentation format. Instead, it should adhere to design principles focused on cognitive load control and task matching. By rationally organizing and configuring multiple explanation formats, designers can support physicians in achieving efficient, controllable, and trustworthy human-AI collaborative diagnosis across varying levels of system reliability.

## 5 Conclusion

This study examined the effects of decision explanation formats and system reliability on human-AI collaborative diagnosis through a simulated diagnostic experiment involving 22 physicians. The results showed that explanation formats were associated with differences in cognitive load and decision efficiency, while no significant effect was detected for diagnostic accuracy; importantly, the effect of explanation format on task completion time varied significantly across system reliability conditions. Specifically, decision tree explanations, characterized by clear hierarchical structures, were associated with lower cognitive load and showed efficiency advantages under specific reliability conditions. Highlighted text explanations, which aligned with physicians’ established clinical reading and reasoning practices, yielded higher levels of subjective trust and user preference. Tabular representations may be more suitable for rapid comparison and reference, whereas flowchart formats should be applied with caution due to their relatively higher cognitive and time costs.

Beyond these empirical findings, the study suggests that the value of explanation formats lies not in maximizing informational completeness, but in achieving an appropriate fit among task complexity, users’ cognitive resources, and system reliability. From a human factors perspective, this work highlights the complementary roles of explanation formats and reliability in medical human-AI collaboration and provides empirical evidence and new insights for conversational AI clinical decision support systems.

## 6 Limitations and future work

Several limitations should be considered when interpreting the present findings. First, the relatively high diagnostic accuracy and constrained response format may have limited the sensitivity of the accuracy measure; therefore, the non-significant finding for diagnostic accuracy should be interpreted cautiously. Second, all participants had clinical experience and completed the tasks in a controlled experimental setting, which may limit the generalizability of the findings to more dynamic and time-constrained clinical environments. In addition, the relatively small sample size may limit the statistical power for detecting small effects and the generalizability of the findings. Finally, the behavioral and subjective measures represented distinct aspects of human–AI collaboration and were analyzed separately; therefore, differences in statistical significance across these measures should not be interpreted as direct statistical differences between the underlying constructs. Future studies could employ larger samples, more challenging diagnostic cases, and more sensitive measures to further examine these effects.

## Data Availability

The raw data supporting the conclusions of this article will be made available by the authors, without undue reservation.
